# Generating multiple alignments on a pangenomic scale

**DOI:** 10.1093/bioinformatics/btaf104

**Published:** 2025-03-17

**Authors:** Jannik Olbrich, Thomas Büchler, Enno Ohlebusch

**Affiliations:** Institute of Theoretical Computer Science, Ulm University, Ulm, 89069, Germany; Institute of Theoretical Computer Science, Ulm University, Ulm, 89069, Germany; Institute of Theoretical Computer Science, Ulm University, Ulm, 89069, Germany

## Abstract

**Motivation:**

Since novel long read sequencing technologies allow for *de novo* assembly of many individuals of a species, high-quality assemblies are becoming widely available. For example, the recently published draft human pangenome reference was based on assemblies composed of contigs. There is an urgent need for a software-tool that is able to generate a multiple alignment of genomes of the same species because current multiple sequence alignment programs cannot deal with such a volume of data.

**Results:**

We show that the combination of a well-known anchor-based method with the technique of prefix-free parsing yields an approach that is able to generate multiple alignments on a pangenomic scale, provided that large-scale structural variants are rare. Furthermore, experiments with real world data show that our software tool PANgenomic Anchor-based Multiple Alignment significantly outperforms current state-of-the art programs.

**Availability and implementation:**

Source code is available at: https://gitlab.com/qwerzuiop/panama, archived at swh:1:dir:e90c9f664995acca9063245cabdd97549cf39694.

## 1 Introduction

A single linear reference is commonly used to represent the human genome in genomic studies and diagnostics. However, there are a lot of differences between the genomes of individuals of the same species and a single reference is unable to cover them. Auton *et al.* (2015) produced a database that includes the genomes of 2504 different humans and the differences between them. Including common variations in the reference gives a more accurate representation of the genomes of a species. We call such a representation the pangenome of the population. It should be pointed out that throughout this paper we use the term “pangenome” in a narrower sense. In a broader sense, the pangenome defines the entire genomic repertoire of a given phylogenetic clade, which may range from species to phylum and beyond. Note that Tettelin *et al.* (2005) coined the term pangenome two decades ago; they evaluated the composition of six strains of *Streptococcus agalactiae*.

A pangenome is often constructed from a reference sequence and a VCF-file containing the variations. Since novel long read sequencing technologies allow for *de novo* assembly of many individuals of a species or population (Porubsky *et al.* 2021), high-quality assemblies are becoming widely available. In this paper, we tackle the problem of generating a multiple alignment of assembled genomes of many individuals of the same species. Such an alignment allows for the detection of SNPs and small-scale structural variants. Of course, a (colinear) multiple alignment cannot capture structural variants such as large-scale inversions, but in many species these are relatively rare. (In future work, we intend to combine our method with techniques that detect such large-scale structural variants.) Up to now, the computation of such a chromosome-scale multiple alignment was not possible because no multiple aligner was able to deal with such a volume of data (cf. Section 7).

Höhl *et al.* (2002) presented Multiple Genome Aligner (MGA), the first software-tool that was able to compute a multiple alignment of closely related genomes. However, MGA was limited to viruses and strains of bacteria (Chain *et al.* 2003). MGA and many other software-tools for aligning multiple genomic DNA sequences use an anchor-based method that is composed of three phases:

1. computation of fragments (segments in the genomes that are identical or very similar),
2. computation of a highest-scoring global chain of colinear nonoverlapping fragments: these are the anchors that form the backbone of the alignment,
3. alignment of the regions between the anchors (either by applying the same method recursively or by applying a different multiple sequence alignment program).

A recent tool that elaborates on this anchor-based method is FMAlign2 (Zhang *et al.* 2024). However, FMAlign2 is still limited to datasets of a few hundred million bases (cf. Section 7).

**Figure 1.**
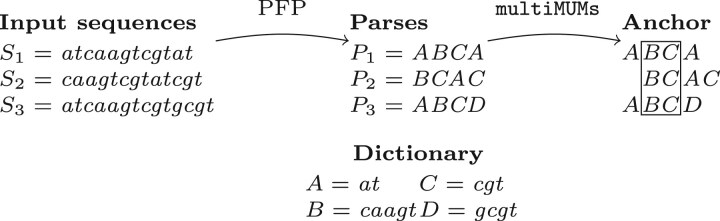
First, the parses of the input sequences will be calculated. Then anchors are determined within the parses.

In this paper, we will show that it is possible to generate a multiple alignment of a set of assembled genomes of the same species, where an assembled genome is a set of contigs. Our new method is depicted in Fig. 1. We combine MGA’s anchor-based method with the technique of prefix-free parsing (PFP), which was introduced by Boucher *et al.* (2019). This technique parses a DNA sequence (a chromosome composed of contigs or a complete chromosome) S into phrases, and two phrases have the same identifier (meta-symbol) if and only if they are identical on the base-level. Thus, the parse P is the sequence of identifiers that gives S if each identifier is replaced with its phrase. The main idea is to first compute anchors on the parse P instead of computing anchors on S, see Fig. 1. Given m DNA sequences from the same chromosome of different individuals (e.g. chromosome 19 of 1000 different humans) as input, our method uses the following phases, which are explained in subsequent sections:

1. Compute the parses of the m sequences.
2. On the parses, compute the backbone of the overall multiple alignment as follows:
  - Compute the generalized suffix array.
  - Find multiMUMs (the fragments).
  - Compute anchors by chaining multiMUMs.
  - Extend anchors on the base-level.
3. Generate an alignment of the gaps. For brevity, we will use the term ‘gap’ instead of ‘region between anchors’ from now on (it should not be confused with a gap in an alignment).
  - While there remains a large gap, apply Phases 2a-2c to the gap and add the fragments in the resulting chain as anchors (here, multiMUMs may be partial).
  - Generate an alignment of the remaining (small) gaps using FMAlign2 and MAFFT (Nakamura *et al.* 2018).

In this way, we are able to generate multiple alignments on a pangenomic scale. It should be pointed out that our new method can only be successful if the chromosomes of individuals from the same species are very similar DNA sequences; in particular large structural chromosomal rearrangements must be rare. Therefore, our software tool PANgenomic Anchor-based Multiple Alignment (PANAMA) is a special purpose multiple sequence alignment program. On the other hand, there is an urgent need for such a program (Liao *et al.* 2023). Experiments with real world data show that our program PANAMA outperforms current state-of-the art programs.

## 2 Prefix-free parsing

Prefix-free parsing is a technique invented by Boucher *et al.* (2019). In the simple version used here, it uses a rolling hash (a hash function where the input is hashed in a window that moves through the input) to divide a string S into substrings, which form the dictionary D. The name prefix-free parsing is justified by the property that no suffix of a string from D is a proper prefix of a suffix of any other string from D (but this property does not play a role in our context). In the following more detailed explanation, we assume the reader to be familiar with the Karp-Rabin-Algorithm (see e.g. Cormen *et al.* 1990). This algorithm uses a sliding window of fixed size w and a hash function KR. For every position i in S, it computes the hash value KR$\left(W_{i}\right)$ of the substring $W_{i}=S[i..i+w-1]$. Since the hash value of $W_{i+1}$ can be computed in constant time from the previous hash value, the parsing algorithm takes only linear time. Given a fixed number p (called modulus; in the Karp-Rabin-Algorithm, the modulus is a prime number, but this is not required in our context), the string $W_{i}$ is called a trigger string if and only if KR$(W_{i})\;\mathrm{mod}\;p=0$. In a left-to-right scan of S, the parsing algorithm breaks S into substrings so that each substring ends with a trigger string (and contains no other trigger string). This gives the dictionary D and the parse P. In P, phrases (elements of D) are represented by their lexicographic rank. More precisely, the phrases are ordered lexicographically and every phrase is identified with its rank in the sorted dictionary (i.e. the ranks serve as meta-symbols). Consequently, the parse P is the sequence of numbers that gives the string S if each number is replaced with its phrase from D. In the example of Fig. 1, the window size is w=1, “t” is used as trigger string, and upper case letters are used as meta-symbols.

## 3 Preliminaries (for computing multiMUMs)

Let S be a string of length n on an ordered alphabet Σ. For 1≤i≤n, S[i] denotes the *character at position* i in S. For i≤j, S[i..j] denotes the *substring* of S starting with the character at position i and ending with the character at position j. Furthermore, $S_{i}$ denotes the ith suffix S[i..n] of S.

The *suffix array* SA of the string S is an array of integers in the range 1 to n specifying the lexicographic ordering of the n suffixes of S, i.e. it satisfies $S_{\mathrm{SA}[1]}<S_{\mathrm{SA}[2]}<\cdots <S_{\mathrm{SA}[n]}$. The suffix array can be built in linear time; we refer to the overview article of Puglisi *et al.* (2007) for suffix array construction algorithms and to Olbrich *et al.* (2024) for newer developments.

Let S be a string of length n having the sentinel character attheend(andnowhereelse).Weassumethat is smaller than any other character. The Burrows and Wheeler transform (Burrows and Wheeler 1994) converts S into the string BWT[1..n] defined by BWT[i]=S[SA[i]−1] for all i with SA[i]≠1 and BWT[i]=$ otherwise.

The suffix array SA is often enhanced with the so-called LCP-array containing the lengths of longest common prefixes between consecutive suffixes in SA. Formally, the LCP-array is an array so that LCP[1]=−1=LCP[n+1] and $\mathrm{LCP}[i]=|\mathrm{lcp}(S_{\mathrm{SA}[i-1]},S_{\mathrm{SA}[i]})|$ for 2≤i≤n, where lcp(u,v) denotes the longest common prefix between two strings u and v. Like the suffix array, the LCP-array can be computed in linear time (Kasai *et al.* 2001). Abouelhoda *et al.* (2004) introduced the concept of lcp-intervals. An interval [i..j], where 1≤i<j≤n, in the LCP-array is called an *lcp-interval of lcp-value* ℓ (denoted by ℓ-[i..j]) if

-
LCP[i]<ℓ ,
-
LCP[k]≥ℓ for all k with i+1≤k≤j,
-
LCP[k]=ℓ for at least one k with i+1≤k≤j,
-
LCP[j+1]<ℓ .

Abouelhoda *et al.* (2004) showed that there is a one-to-one correspondence between the set of all lcp-intervals and the set of all internal nodes of the suffix tree of S (we assume a basic knowledge of suffix trees). Consequently, there are at most n−1 lcp-intervals for a string of length n.

Let $S^{1},S^{2},\ldots ,S^{m}$ be strings of sizes $n_{1},n_{2},\ldots ,n_{m}$, respectively. We are interested in the lexicographic order of all suffixes


$$S_{1}^{1},\ldots ,S_{n_{1}}^{1},S_{1}^{2},\ldots ,S_{n_{2}}^{2},\ldots ,S_{1}^{m},\ldots ,S_{n_{m}}^{m}$$

of these strings. Note that two suffixes $S_{p}^{j}$ and $S_{q}^{k}$ with j≠k may coincide, i.e. $S_{p}^{j}=S_{q}^{k}$ is possible. (In this case, it is natural to demand that the suffix with the smaller superscript shall appear before the suffix with the larger superscript.) Because the strings may share identical suffixes, we use m pairwise distinct characters ${\#}_{1},{\#}_{2},\ldots ,{\#}_{m}$ to tell the suffixes apart. To be precise, for each j with 1≤j≤m, we obtain the string $S^{j}{\#}_{j}$ of length $n_{j}+1$ by appending the special character ${\#}_{j}$ to $S^{j}$. This ensures that each suffix can uniquely be assigned to one of the m strings: if the suffix ends with ${\#}_{j}$, then it belongs to $S^{j}$. If we assume that ${\#}_{1}<{\#}_{2}<\ldots <{\#}_{m}$ and that all other characters in the alphabet Σ are larger than these symbols, then the suffixes of the strings $S^{1}{\#}_{1},S^{2}{\#}_{2},\ldots ,S^{m}{\#}_{m}$ are not only pairwise distinct, but we also have $S_{p}^{j}{\#}_{j}<S_{q}^{k}{\#}_{k}$ if and only if either $S_{p}^{j}<S_{q}^{k}$ or $S_{p}^{j}=S_{q}^{k}$ and j<k. In the following, we tacitly assume that every string $S^{j}$ (1≤j≤m) is terminated with the character ${\#}_{j}$, so its size is $n_{j}+1$.

The *generalized suffix array* (GSA) of the strings $S^{1},S^{2},\ldots ,S^{m}$ consists of *two* arrays of size $n=m+\sum_{j=1}^{m}n_{j}$, the *document array* DA and the array SA, having the following properties:

- For every suffix $S_{k}^{j}$, there is an index i so that j=DA[i] and k=SA[i].
-
$S_{\mathrm{SA}[i]}^{\mathrm{DA}[i]}<S_{\mathrm{SA}[i+1]}^{\mathrm{DA}[i+1]}$ for all i with 1≤i≤n−1.

**Figure 2.**
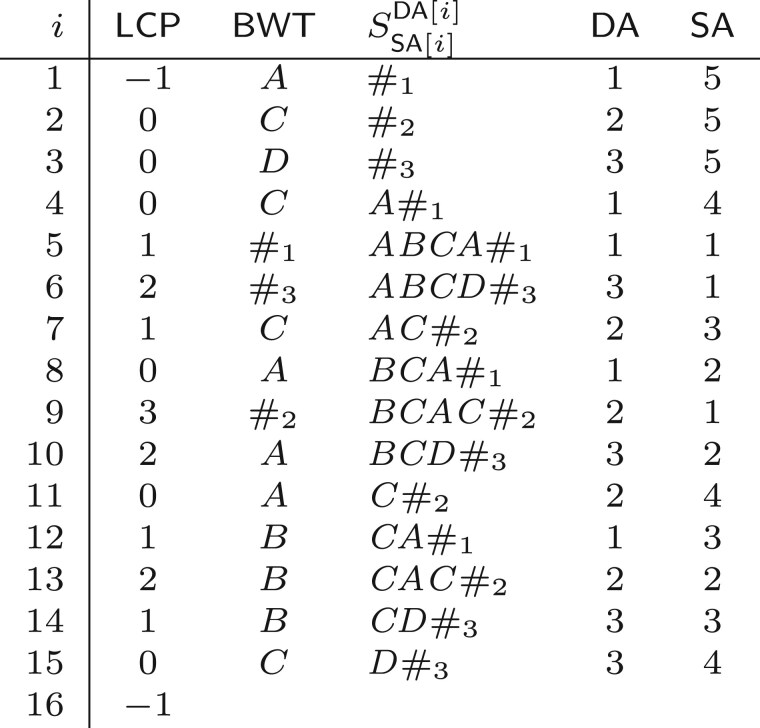
The GSA of the strings $S^{1}=\mathrm{ABCA}{\#}_{1}$, $S^{2}=\mathrm{BCAC}{\#}_{2}$, and $S^{3}=\mathrm{ABCD}{\#}_{3}$ (derived from the parses of Fig. 1) consists of the two arrays DA and SA. The enhanced GSA includes the corresponding LCP-array (note that the BWT can easily be computed on the fly).

In other words, the arrays DA and SA specify the lexicographic order of all the suffixes of the m strings. An example can be found in (the two rightmost columns of) Fig. 2, which also shows the corresponding LCP-array and BWT. We will call the combination of the GSA with the LCP-array the *enhanced* GSA of $S^{1},S^{2},\ldots ,S^{m}$. Louza *et al.* (2017) have shown that the (enhanced) GSA can be constructed in O(n) time with only one special character instead of the m special characters ${\#}_{1},{\#}_{2},\ldots ,{\#}_{m}$ (this is advantageous because it keeps the alphabet small). However, it is conceptually easier to use m special characters instead of one.

## 4 Computation of multiMUMs

In order to determine anchors (on the parse-level or on the base-level), we must first compute fragments and then a highest-scoring global chain of colinear nonoverlapping fragments. MGA (Höhl *et al.* 2002) as well as FMAlign2 (Zhang *et al.* 2024) use multiMEMs as fragments, but like parsnp (Treangen *et al.* 2014) we use multiMUMs instead. This is because the explanation of how multiMUMs can be computed is much easier to understand and preliminary experiments showed that multiMUMs are equally good. Roughly speaking, a multiMUM is a string ω occurring exactly once in each of the sequences $S^{1},\ldots ,S^{m}$ with the property that ω cannot simultaneously be extended in all sequences (on either end) without incurring a mismatch. The formal definition reads as follows.

Definition 1. *A multiple exact match in the sequences* $S^{1},\ldots ,S^{m}$  *is an* (m+1)*-tuple* $\left(\ell ,p_{1},\ldots ,p_{m}\right)$  *with* ℓ>0  *and* $1\leq p_{k}\leq n_{k}-\ell +1$  *(*1≤k≤m*) so that* $S^{i}[p_{i}..p_{i}+\ell -1]=S^{j}[p_{j}..p_{j}+\ell -1]$  *for all* i,j∈{1,…,m}*. In words, the length* ℓ  *substrings of* $S^{1},\ldots ,S^{m}$  *starting at the positions* $p_{1},\ldots ,p_{m}$  *coincide. A multiple exact match is left-maximal if for at least one pair* (i,j)  *we have* $S^{i}[p_{i}-1]\neq S^{j}[p_{j}-1]$  *(for* k∈{1,…,m}*, we define* $S^{k}[p_{k}-1]={\#}_{k}$  *if* $p_{k}=1$*, see the definition of the BWT). It is right-maximal if for at least one pair* (i,j)  *we have* $S^{i}[p_{i}+\ell ]\neq S^{j}[p_{j}+\ell ]$*. A multiple exact match is maximal if it is left-maximal and right-maximal. A multiple maximal exact match is also called multiMEM. A multiMEM* $\left(\ell ,p_{1},\ldots ,p_{m}\right)$  *is a multiMUM (multiple maximal unique match) if for all* i  *with* 1≤i≤m  *the string* $S^{i}[p_{i}..p_{i}+\ell -1]$  *occurs exactly once in the sequence* $S^{i}$.

If there is a long multiMUM  $\left(\ell ,p_{1},\ldots ,p_{m}\right)$ in the sequences $S^{1},\ldots ,S^{m}$, then it is very likely that the identical substrings $S^{1}[p_{1}..p_{1}+\ell -1],\ldots ,S^{m}[p_{m}..p_{m}+\ell -1]$ appear one below the other in a multiple alignment of $S^{1},\ldots ,S^{m}$. In other words, the multiMUM serves as a potential anchor. The next lemma tells us how multiMUMs can efficiently be computed.

Lemma 2. *There is a one-to-one correspondence between the set of all multiMUMs and the set of all lcp-intervals* ℓ*-*[lb..rb]  *in the enhanced GSA of* $S^{1},\ldots ,S^{m}$  *satisfying*
 

1. rb−lb+1=m.
2. DA[i]=DA[j]  *for all pairs* (i,j)  *with* lb≤i<j≤rb.
3. BWT[i]=BWT[j]  *for at least one pair* (i,j)  *with* lb≤i<j≤rb.

According to the preceding lemma we can compute multiMUMs as follows: Enumerate all lcp-intervals and for each lcp-interval ℓ-[lb..rb] of size m check whether the m indices in [lb..rb] satisfy conditions (2) and (3). In the example of Fig. 2, 2-[8.0.10] is the only lcp-interval of size 3. This interval fulfills conditions (2) and (3) and hence it corresponds to the multiMUM (2,2,1,2), which is the anchor in Fig. 1.

It is well known that all lcp-intervals can be enumerated in O(n) (Kasai *et al.* 2001, Abouelhoda *et al.* 2004). The enumeration algorithm is shown in the Supplementary Material. The proof of Lemma 2 and an alternative method for computing multiMUMs can also be found in the Supplementary Material.

## 5 Fragment-chaining and extension of anchors

To find anchors, one must find a highest-scoring global chain of colinear nonoverlapping fragments. In our context, a fragment f is a multiMUM  $\left(\ell ,p_{1},\ldots ,p_{m}\right)$. We associate a weight with each fragment, denoted by f.weight. If f is a multiMUM on the base-level, then we set f.weight=ℓ. If f is a multiMUM on the parse-level, then f.weight is the number of bases that are obtained by replacing the identifiers (meta-symbols) in $S^{k}[p_{k}..p_{k}+\ell -1]$ with their phrases from the dictionary.

**Figure 3.**
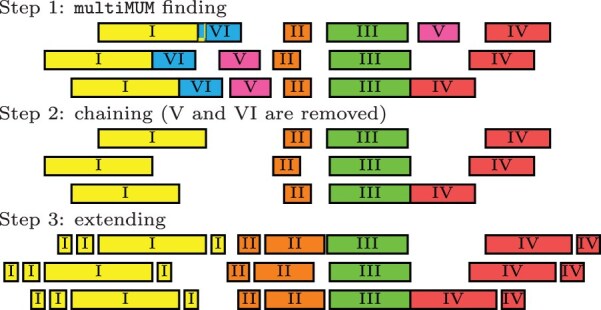
Schematic construction of our backbone. Step 1 initially runs on the parse-level. Note that the situation of multiMUM V is highly unlikely to occur and would likely indicate a transposition. This case is included solely to exhaustively display the reasons why a multiMUM may be excluded from the chain in Step 2. Step 3 is done on the base-level, it is explained at the end of Section 5. Note that the Steps 1–3 here correspond to Phases 2b–2d (see Section 1).

Roughly speaking, two fragments f and $f^{\prime }$ are *colinear* if their order is the same in all of the sequences. In Step 1 of Fig. 3, e.g. the fragments I and II are colinear, but II and V are not. Two fragments *overlap* if their segments overlap in one of the sequences (in Fig. 3, the fragments I and VI are overlapping, while I and II are nonoverlapping).

We define a binary relation ≪ on the set of fragments so that $f\ll f^{\prime }$ if and only if f and $f^{\prime }$ are colinear and nonoverlapping.

Definition 3. *Let* $f=(\ell ,p_{1},\ldots ,p_{m})$  *and* $f^{\prime }=(\ell^{\prime },p_{1}^{\prime },\ldots ,p_{m}^{\prime })$  *be two fragments. We define* $f\ll f^{\prime }$  *if and only if* $p_{k}+\ell -1<p_{k}^{\prime }$  *for all* k  *with* 1≤k≤m*. We then say that* f  *precedes* $f^{\prime }$.

*A chain* C  *of colinear nonoverlapping fragments (“chain” for short) is a sequence of fragments* $f_{1},f_{2},\ldots ,f_{j}$  *so that* $f_{i}\ll f_{i+1}$  *for all* i  *with* 1≤i<j*. The score of* C  *is* $\mathrm{score}(C)=\sum_{i=1}^{j}f_{i}.\;\mathrm{weight}$.

*Given* m  *weighted fragments, the global fragment-chaining problem is to determine a chain of highest score (called optimal global chain in the following) starting at the origin 0 and ending at terminus t. (The origin* 0=(0,0,…,0)  *and the terminus* $t=(0,n_{1}+1,\ldots ,n_{m}+1)$  *are artificial fragments with weight* 0*. Note that* 0≪f≪t  *for every fragment* f  *with* f≠0  *and* f≠t*.)*

Let $f^{\prime }.\;\mathrm{score}$ be defined as the maximum score of all chains starting at 0 and ending at $f^{\prime }$. Then $f^{\prime }.\;\mathrm{score}$ can be expressed by the recurrence: 0.score=0 and


$$f^{\prime }.\;\mathrm{score}=f^{\prime }.\;\mathrm{weight}+\max\{f.\;\mathrm{score}|f\ll f^{\prime }\}$$

A dynamic programming algorithm based on this recurrence takes $O(mk^{2})$ time to compute an optimal global chain, where k is the number of fragments.

We can use the fact that we expect an optimal global chain to contain almost all of the fragments to reduce the expected time complexity to O(k log k+mk): We sort the fragments by increasing position in e.g. the first sequence and process them in this order. Moreover, we maintain the already processed fragments in an array sorted by score. For each fragment $f^{\prime }$ we search for its predecessor of highest score as follows: We consider the already processed fragments (starting with the one with the highest score yet) and pick the first fragment which is actually a predecessor of $f^{\prime }$. For almost all fragments, the number of other fragments checked will be one and the fragment will be inserted at the end of the array. Of course, this heuristic does not reduce the worst case time complexity, but it works very well in our context.

The fragments (multiMUMs) in an optimal global chain on the parse-level are the initial anchors of the multiple alignment of the m sequences. Such an anchor (a multiMUM of the parses) cannot be extended on the parse-level, but in most cases it can be extended on the base-level. This is because the phrases corresponding to two different meta-symbols may share a common suffix (so that a left extension on the base-level may be possible) or prefix (ditto for a right extension). If we replace each meta-symbol in a multiMUM on the parse-level with its corresponding phrase from the dictionary, then we obtain a multiple exact match on the base-level. We simultaneously extend it base-by-base to the left (and right, respectively) in each of the m sequences until a mismatch occurs. Since such a mismatch is most likely a SNP, we try to further extend it by the same procedure. This iterative extension ends when <10 bases match exactly (simultaneously in all of the sequences) beyond one mismatch. This is illustrated in Step 3 of Fig. 3: The anchors I, II, and IV are extended on the base-level (anchor I is extended twice to the left). After this extension step, we have the final anchors (the backbone) of the overall multiple alignment.

## 6 Generating alignments of the gaps

After the computation of the anchors, we need to fill the gaps between them. (Note that this can trivially be parallelized because the alignments of two gaps are independent of each other.) For small gaps, we can simply use another multiple-sequence aligner [we use FMAlign2 (Zhang *et al.* 2024) and MAFFT (Nakamura *et al.* 2018)]. However, when there are large gaps, e.g. because the sequences are incomplete, those other programs take very long to run.

For this reason, we try to fill the gaps using *partial*multiMUMs. A partial multiMUM is a multiMUM of a nonempty subset S of the sequences. For convenience, we denote it by $\left(\ell ,p_{1},\ldots ,p_{m}\right)$, where $p_{i}$ is the position of the multiMUM in sequence i if this sequence is in S and $p_{i}=⊥$ otherwise (where ⊥ is a special value indicating absence). We say the partial multiMUM  *occurs* in sequence i if and only if $p_{i}\neq ⊥$, and for all i∈{1,…,m} with $p_{i}\neq ⊥$ it is required that the string $S^{i}[p_{i}..p_{i}+\ell -1]$ occurs exactly once in $S^{i}$. Note that this definition does not require the converse to be true (i.e. if the string $S^{i}[p_{i}..p_{i}+\ell -1]$ occurs exactly once in sequence i, the partial multiMUM must not necessarily occur in sequence i). This is because our algorithm for chaining partial multiMUMs may discard the occurrence of a partial multiMUM in a sequence in order to increase the overall score of the chain (although this is unlikely).

**Figure 4.**
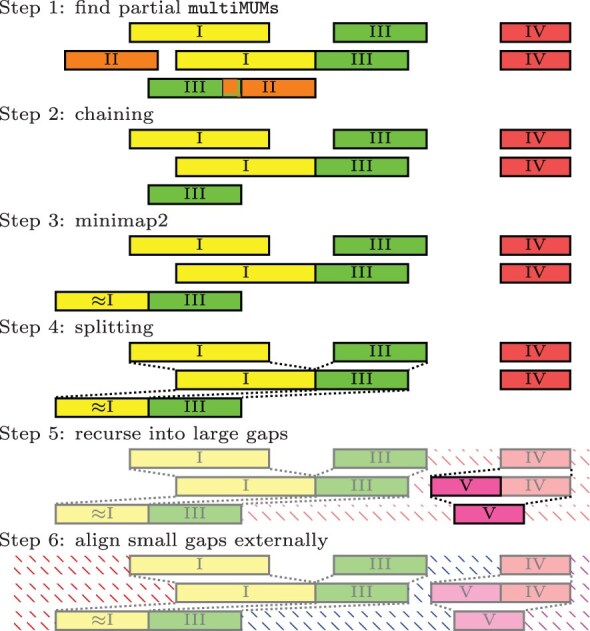
Schematic filling of a large gap. Note that it is possible to find multiMUMs that span all sequences (such as III) which were not discovered when considering all sequences. This can happen when the same string also appears somewhere outside the large gap, or because we compute multiMUMs on the base-level instead of on the parse-level.

In what follows, we describe our process of completing the alignment (Phase 3 in the Section 1) in the six steps that are visualized in Fig. 4. The Steps 1–5 correspond to Phase 3a and Step 6 equals Phase 3b.

### Step 1: find partial multiMUMs

The first step is to find partial multiMUMs. By definition, this can be achieved using a trivial modification (replacing rb−lb+1=m with rb−lb+1≤m in Lemma 2) of the algorithm for finding multiMUMs. If the gap is sufficiently large, we work on the parse-level, otherwise we work on the base-level. If a previous attempt on the parse-level of this gap did not yield any new anchors, we also switch to the base-level. For algorithmic reasons (see below), we require each partial multiMUM to occur in more than half of the sequences.

### Step 2: chain partial multiMUMs

As in the computation of the backbone, we chain the fragments (partial multiMUMs). However, the weight of a fragment must now also incorporate the number of sequences the fragment occurs in. As new weight, we use the length of the fragment multiplied by the number of sequences it occurs in [i.e. the total number of bases it covers, as does FMAlign2 (Zhang *et al.* 2024)]. Unfortunately, chaining partial fragments appears to be fundamentally more difficult than chaining fragments occurring in all sequences. This is due to the fact that the ≪-relation on partial fragments is not transitive anymore. [All (polynomial-time) chaining algorithms known to us rely on this transitivity.] To circumvent this issue, we use a heuristic. Specifically, we select k sequences and then chain only those partial multiMUMs occurring in all of these k sequences. The selected sequences are those with the most bases covered by the found partial multiMUMs. We do this for all $k\in \{\left\lfloor \frac{m}{2}\right\rfloor +1,\ldots ,m\}$, and then choose the resulting chain with the most bases covered. The restriction $k>\frac{m}{2}$ is required for every pair of partial multiMUMs to be either incompatible (i.e. overlapping or not colinear) or comparable with ≪, and thus ensures that the anchors can be totally ordered.

The partial multiMUMs in the resulting “sub-chain” may have had occurrences in the m−k other sequences, which should of course not be discarded. Thus, we add them to the chain with a simple variant of the well-known algorithm for the heaviest increasing subsequence (HIS) (Jacobson and Vo 1992) (since the order of the partial multiMUMs in the sub-chain is fixed, this can be done separately for each sequence).

### Step 3: extend partial fragments with minimap2

**Figure 5.**
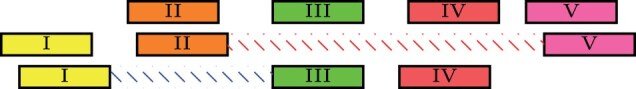
Since we require that each anchor occurs in more than half of the sequences, they can be linearly ordered (I≪II≪III≪IV≪V). Consider the gap in the second sequence between II and V. The anchors III and IV are between II and V in the linear order, thus we try to map them (the string they represent) to this gap using minimap2. Similarly, we would try to map II to the gap between I and III in the third sequence, I to the gap before II in the first sequence, and V to the third sequence after IV.

After computing such a sub-chain, we try to extend each resulting partial fragment to the sequences it does not occur in using the long read mapper minimap2 (Li 2021). This is illustrated in Fig. 5.

Recall that the fragments are linearly ordered because each occurs in more than half the sequences. Let those fragments be $f_{1}\ll \ldots \ll f_{k}$. We consider each gap in each sequence separately. For such a gap, we determine the two fragments $f_{i}$ and $f_{j}$ (1≤i<j≤k) that bound it and try to map the fragments between them ($\left\{f_{i+1},\ldots ,f_{j-1}\right\}$) to this gap using minimap2. The matches reported by minimap2 are then chained using the same HIS variant as above.

Now the fragments in the sub-chain are not changed anymore and are considered anchors.

### Steps 4–6: complete alignment

After adding the new anchors, we again try to split the large gap into smaller gaps, which are independent of each other.

If a resulting gap is sufficiently small, we use FMAlign2 to align it, otherwise we recurse. It may be the case that, after splitting, a gap still contains some anchors. (This happens when anchors occur in too few sequences and do not lead to a split.) These anchors are kept, and we ensure that chains in lower recursion levels are consistent with them.

If there is a sequence in a large gap that cannot be aligned to the other sequences via (partial) multiMUMs or minimap2, this gap cannot be split. However, since we can assume that there is no match anyway (because neither minimap2 nor we could find one), we remove this sequence from this gap before recursing and “align” it immediately after the gap. This is only done when there are anchors exceeding a given length that span the other sequences.

## 7 Experimental results

We implemented our algorithm in an experimental tool, called PANAMA, in C++. The source code is publicly available (https://gitlab.com/qwerzuiop/panama). For the construction of enhanced suffix arrays we used the SDSL-lite library (Gog *et al.* 2014).

We evaluated our implementation on two datasets, namely data from the 1000 Genomes Project (http://dolomit.cs.tu-dortmund.de/tudocomp/chr19.1000.fa.xz archived at https://www.uni-ulm.de/in/theo/research/seqana/panama) (56 GiB) and data from the draft human pangenome reference (https://s3-us-west-2.amazonaws.com/human-pangenomics/index.html?prefix=working) (21 GiB) (Liao *et al.* 2023). The generated alignments are publicly available (https://www.uni-ulm.de/in/theo/research/seqana/panama).

The first dataset stems from Boucher *et al.* (2021) and contains 1000 human haplotypes of chromosome 19. The sequences within this set all have a length of about 59 million base pairs. It can be viewed as a set of complete telomere-to-telomere assemblies (there is no missing data).

The second dataset contains the phased, diploid assemblies of 47 genetically diverse humans. In order to determine the ordering and orientation of the contigs of the assemblies, we utilized the mapping software gedmap (Büchler *et al.* 2023) as a long read mapper. For each phased assembly A, we split each contig $c_{A}$ of A into 500 bp long reads and mapped these to the reference genome hs37d5 including the variants from the 1000 genome project (Auton *et al.* 2015). The resulting alignments were chained and the best chain was used to map $c_{A}$ to the reference. In most cases, the best chain distinctly revealed the position and orientation of $c_{A}$ relative to the reference. When a contig $c_{A}$ could not be placed, it was excluded from further processing. We selected all contigs of A that were aligned to chromosome 1 (the largest human chromosome) and sorted them by their position. If necessary, we replaced them with their reverse complement. The concatenation of these ordered contigs (separated by special symbols) was used as the (partial) DNA sequence of chromosome 1 of assembly A. In our experiment, we included all sequences (obtained in this way) that covered at least 80% of chromosome 1, which was the case for 92 out of the 94 assemblies. It is thus not surprising that the lengths of the sequences in this set range from 203 to 250 million base pairs. This means that we allowed up to 20% missing data per sequence, which is an additional challenge when generating the alignment.

For filling the small gaps (at most $10^{7}$ bases), we used FMAlign2 (Zhang *et al.* 2024) with HAlign3 (Tang *et al.* 2022) as backend, and fell back to MAFFT (Nakamura *et al.* 2018) in case FMAlign2 produced no output (FMAlign2 crashes on some inputs. This affects mostly simple cases, e.g. where all sequences are prefixes of the same string.). We accepted matches from minimap2 with a MAPQ (“mapping quality”) value of at least 30 (This corresponds to a likelihood of the mapping being wrong of at most 0.01%. Note that after accepting matches, we still chain them.). Since the modulus used in PFP for determining trigger strings influences the expected length of the phrases, we evaluated our program PANAMA with different moduli (20 and 100). The window size for the KR-hashing was always 10, because it seems to not significantly affect the properties of the parsing as long as it is sufficiently large (Boucher *et al.* 2019). Note that a window size of 10 is also the default used in Boucher *et al.* (2019). The modulus for PFP in large gaps was always 20.

All experiments were conducted on a Linux-6.5.0 machine with an AMD EPYC 7742 (64 cores, 128 threads) processor and 256GB of RAM. We compiled PANAMA with GCC 11.4.0 with optimization flags -O3 -funroll-loops -march=native -DNDEBUG. The reported wall clock time was measured with the utility GNU Time. The reported RAM usage was calculated by measuring the maximum resident system RAM usage during execution and subtracting the resident system RAM usage before/after execution (Since FMAlign2 and PANAMA execute other external programs, measuring RAM usage with e.g. GNU Time is not sufficient.). The results of our experiments can be seen in Tables 1 and 2. By far the most memory hungry phase is Phase 3, which invokes external aligners in parallel (the external aligners are responsible for most of the maximum RAM usage here). Therefore, the memory usage of PANAMA could greatly be reduced by reducing the number of concurrent threads.

**Table 1.** Wall clock time in minutes of the individual phases for our two test sets and two PFP-moduli, and the maximum RAM usage in GiB.^a^

Dataset:modulus	c19:100	c19:20	c1:100	c1:20
1: Prefix-free parsing	34	47	15	20
2: Compute backbone	28	125	9	35
3: Complete alignment	22	23	40	40
Total	98	195	64	95
RAM	64	69	51	111

a The numbering of the phases refers to the overview in Section 1.

**Table 2.** Evaluation of the generated alignments.^a^

Dataset:modulus	c19:100	c19:20	c1:100	c1:20
No. of anchors after 2c	88 788	305 480	336 206	640 794
No. of extensions at 2d	335 644	509 986	661 532	712 606
Coverage in percent:				
After 2c (chaining)	19.01	49.30	23.28	49.27
After 2d (extension)	65.84	92.57	63.98	70.32
In final alignment	100	100	81.38	81.38
Identity in percent:	97.93	98.15	75.93	75.90

a The number of anchors equals the number of multiMUMs in the chain computed in Phase 2c. The number of extensions equals the number of left or right extension of anchors beyond a mismatching column. This can also be seen as the number of potentially detected SNPs at this phase. Percent coverage is the number of bases that are included in our alignment relative to the overall number of bases. Percent identity is the number of 100% identity columns (i.e. columns without gaps in which all bases are identical) relative to the alignment length. The centromeric region of chromosome 1 is included in all measurements but excluded from the computation of the identity.

There is no data missing in the chromosome 19 dataset, thus the gaps in the backbone (i.e. after extension 2d) are small. Tables 1 and 2 show that PANAMA can generate a high quality multiple alignment of 1000 haplotypes of chromosome 19 within hours. (Of course, such a high quality alignment is possible only if the input sequences are very similar, which is the case here.)

In contrast, on the chromosome 1 dataset, the number of bases per sequence varies greatly because we had to use contigs instead of whole chromosomes. This variance leads to large gaps in the backbone. In particular, there is one large gap for which PANAMA could not produce a reasonable alignment. This gap is located around the centromere (Logsdon *et al.* 2024), starting at around base 1.03 × 10^8^ and ending at around base 1.36 × 10^8^. Since the reference genome is not part of our dataset, the stated positions are estimated as the number of bases preceding the gap. The start position of the gap varies by less than $10^{6}$ bases, but the number of bases per sequence in this gap ranges from $1.2\;\times \;10^{7}$ to $5.7\;\times \;10^{7}$. The reported end position is the start position plus the median of the number of bases in the sequences in this gap. The centromere is known to be particularly difficult to align as it is highly repetitive (Logsdon *et al.* 2024). Since PANAMA was unable to generate a reasonable alignment of the centromeric region, the coverage is “only” 81%, see Table 2.

Note that the suffix array construction algorithms in the SDSL-lite library (DivSufSort and qsufsort) are not the fastest ones that exist [see e.g. Olbrich *et al.* (2024) for a recent comparison] and are not parallelized. Moreover, the library is generally focused on low memory usage instead of runtime. Therefore, we conjecture that the performance (especially of Phase 2a “compute GSA”) of our implementation can be significantly improved.

We also ran FMAlign2 (with HAlign3 as backend), HAlign3, and MAFFT v7.490 (all with 128 threads and otherwise default options) on sets of chromosome 19 haplotypes. The results are shown in Table 3. FMAlign2 is able to compute the alignment of 3 to 6 haplotypes in the time PANAMA requires for all 1000 (depending on the modulus used), but is not suitable for the whole chromosome 19 dataset. On these datasets, HAlign3 is much faster than FMAlign2 but still slower than PANAMA. HAlign3 crashed on cases with >26 haplotypes with a “NullPointerException.” MAFFT already crashed on two chromosome 19 haplotypes with a “TOO MANY SEGMENTS” error.

**Table 3.** Results of FMAlign2, HAlign3, and PANAMA (with PFP modulus 100) on a few haplotypes of chromosome 19.

(a) FMAlign2 running on small sets of chromosome 19 haplotypes.						
Number of haplotypes	2	3	4	5	6	7
Wall clock time (min)	36	78	123	167	216	270
RAM (GiB)	19	27	35	43	51	59

(b) HAlign3 running on small sets of chromosome 19 haplotypes.					
Number of haplotypes	2	4	16	24	26
Wall clock time (min)	0.6	0.7	1.0	1.6	2.4
RAM (GiB)	10.0	12.2	26.5	32.2	32.5

(c) PANAMA running on small sets of chromosome 19 haplotypes.					
Number of haplotypes	2	4	16	24	26
Wall clock time (min)	0.2	0.4	1.2	1.7	1.8
RAM (GiB)	0.4	0.5	0.8	1.0	1.2

We compared the alignments for small numbers of Chromosome 19 haplotypes generated by PANAMA with those generated by FMAlign2 and HAlign3 using the average sum-of-pairs edit distance (SP-value), i.e. the sum of the pairwise Levenshtein distances (Levenshtein 1966) divided by the number of sequences. Consequently, a smaller SP-value is better. The results are shown in Table 4.

**Table 4.** SP-value of the alignments generated by FMAlign2, HAlign3, and PANAMA (with PFP modulus 100) on varying numbers of chromosome 19 haplotypes.^a^

Number of haplotypes	2	3	4	16	24
FMAlign2	8.96	5.78	4.64	–	–
HAlign3	0.85	1.05	1.19	1.29	69.0
PANAMA	0.79	0.87	0.90	1.02	1.06

a The displayed SP-value is scaled by a factor of $10^{-5}$. Because the tools handle nonbase characters (e.g. N) differently, we removed nonbase characters from the sequences before aligning them.

In Section 1, we hypothesized that our approach only works for very similar sequences (e.g. chromosomes from human individuals). To test this hypothesis, we evaluated our program on strains of *Escherichia coli*, which are known to be very diverse (Lukjancenko *et al.* 2010) (https://www.ncbi.nlm.nih.gov/datasets/genome/?taxon=562&typical_only=true&assembly_level=3:3, last accessed: 18.11.2024). The results can be seen in Table 5. Note that for many pairs of sequences, a pairwise alignment corresponding to the Levenshtein distance only has approximately 70% identity columns. The Levenshtein distance was computed with A*PA2 (Koerkamp 2024) and the corresponding alignment was generated from the resulting CIGAR string. As a result, the percentage of nucleotides in blocks after Phase 2d quickly drops as the number of sequences increases. The large edit distances between pairs of genomes is (in part) due to large inversions [up to approximately 10%, identified manually using MUMmer4 (Marçais *et al.* 2018)] and varying sequence lengths (up to 20% difference). Because of this, there is no sensible multiple sequence alignment for some of the gaps between blocks and the external aligners consequently take very long to complete them.

**Table 5.** PANAMA running on various numbers of *Escherichia coli* genomes.

Number of haplotypes	2	3	4	8	16
Coverage after 2d in %	58.7	52.1	42.3	32.4	27.0
Identities (final) in %	62.9	55.8	42.9	31.6	30.5
Running time (min)	1.4	1.6	195.7	524.3	864.4

## 8 Conclusions and future work

The experiments with human genomes showed that our tool PANAMA can very efficiently generate a high-quality pangenomic-scale multiple alignment of assembled genomes of the same species. The construction of such a global multiple alignment can be seen as a first step in generating a pangenome graph (Eizenga *et al.* 2020, Baaijens *et al.* 2022), but it is interesting in its own right. In the near future, we plan to extend our work in such a way that it also generates a pangenome graph from a high-quality multiple alignment [and then we can compare with related work on pangenome graphs (Garrison *et al.* 2018, Armstrong *et al.* 2020, Li *et al.* 2020, Garrison *et al.* 2024, Hickey *et al.* 2024); at the moment a reasonable comparison is not possible].

On the other hand, the experiments with E. coli genomes, in which many inversions are present, showed the limits of our approach. (More results with simulated structural variants can be found in the Supplementary Material.) It could be argued that in the presence of large-scale chromosomal rearrangements, a (colinear) multiple alignment of the chromosomes does not make sense at all. In that case, a pangenome graph is certainly a better representation of the genomes. We also plan to extend our work in such a way that it can deal with large-scale structural variants. Our approach can be modified to address this very important topic as follows: instead of computing a multiple alignment, it should be possible to use the syntenic regions as the backbone of a pangenome graph. Inversions and transpositions can be detected before [by pairwise whole-genome alignments with tools such as MUM&Co (O’Donnell and Fischer 2020)] or during the construction of the full pangenome graph. Moreover, programs for pangenome graph generation can be used to deal with the remaining (relatively small) gaps between anchors.

## Supplementary Material

btaf104_Supplementary_Data

## Data Availability

Data used in this paper is publicly available at https://www.uni-ulm.de/in/theo/research/seqana/panama/.

## Notes

A preliminary version of this article appeared in Olbrich *et al.* (2025).

## References

[btaf104-B1] Abouelhoda M , Kurtz S, Ohlebusch E. Replacing suffix trees with enhanced suffix arrays. J Discret Algorithms 2004;2:53–86.

[btaf104-B2] Armstrong J , Hickey G, Diekhans M et al Progressive cactus is a multiple-genome aligner for the thousand-genome era. Nature 2020;587:246–51. 33177663 10.1038/s41586-020-2871-y PMC7673649

[btaf104-B3] Auton A , Brooks LD, Durbin RM et al; The 1000 Genomes Project Consortium. A global reference for human genetic variation. Nature 2015;526:68–74. 26432245 10.1038/nature15393 PMC4750478

[btaf104-B4] Baaijens JA , Bonizzoni P, Boucher C et al Computational graph pangenomics: a tutorial on data structures and their applications. Nat Comput 2022;21:81–108. 36969737 10.1007/s11047-022-09882-6 PMC10038355

[btaf104-B5] Boucher C , Gagie T, Kuhnle A et al Prefix-free parsing for building big BWTs. Algorithms Mol Biol 2019;14. https://almob.biomedcentral.com/articles/10.1186/s13015-019-0148-5#citeas 10.1186/s13015-019-0148-5 PMC6534911 31149025

[btaf104-B6] Boucher C, Gagie T, Tomohiro I et al PHONI: streamed matching statistics with multi-genome references. In: *Data Compression Conference*. Snowbird, UT, USA, IEEE, 2021, 193–202. 10.1109/dcc50243.2021.00027 PMC8583545 34778549

[btaf104-B7] Büchler T , Olbrich J, Ohlebusch E. Efficient short read mapping to a pangenome that is represented by a graph of ED strings. Bioinformatics 2023;39:btad320. 37171844 10.1093/bioinformatics/btad320 PMC10232250

[btaf104-B8] Burrows M , Wheeler D. A block-sorting lossless data compression algorithm. Research Report 124, Digital Systems Research Center. 1994.

[btaf104-B9] Chain P , Kurtz S, Ohlebusch E et al An applications-focused review of comparative genomics tools: capabilities, limitations and future challenges. Brief Bioinform 2003;4:105–23. 12846393 10.1093/bib/4.2.105

[btaf104-B10] Cormen T , Leiserson C, Rivest R. Introduction to Algorithms. Cambridge, MA: MIT Press, 1990.

[btaf104-B11] Eizenga JM , Novak AM, Sibbesen JA et al Pangenome graphs. Annu Rev Genomics Hum Genet 2020;21:139–62. 32453966 10.1146/annurev-genom-120219-080406 PMC8006571

[btaf104-B12] Garrison E , Sirén J, Novak AM et al Variation graph toolkit improves read mapping by representing genetic variation in the reference. Nat Biotechnol 2018;36:875–9. 30125266 10.1038/nbt.4227 PMC6126949

[btaf104-B13] Garrison E , Guarracino A, Heumos S et al Building pangenome graphs. Nat Methods 2024;21:2008–12. 39433878 10.1038/s41592-024-02430-3

[btaf104-B14] Gog S, Beller T, Moffat A et al From theory to practice: plug and play with succinct data structures. In: *Symposium on Experimental Algorithms*. Berlin, Heidelberg: Springer, 2014, 326–37.

[btaf104-B15] Hickey G , Monlong J, Ebler J et al; Human Pangenome Reference Consortium. Pangenome graph construction from genome alignments with Minigraph-Cactus. Nat Biotechnol 2024;42:663–73. 37165083 10.1038/s41587-023-01793-w PMC10638906

[btaf104-B16] Höhl M , Kurtz S, Ohlebusch E. Efficient multiple genome alignment. Bioinformatics 2002;18:S312–20. 12169561 10.1093/bioinformatics/18.suppl_1.s312

[btaf104-B17] Jacobson G , Vo K. Heaviest increasing/common subsequence problems. In: Apostolico A, Crochemore M, Galil Z, Manber U (eds) Combinatorial Pattern Matching. Berlin, Heidelberg: Springer, 1992, 52–66.

[btaf104-B18] Kasai T, Lee G, Arimura H et al Linear-time longest-common-prefix computation in suffix arrays and its applications. In: Combinatorial Pattern Matching. Berlin, Heidelberg: Springer, 2001, 181–92.

[btaf104-B19] Koerkamp RG. APA2: up to 19× faster exact global alignment. In: *Workshop on Algorithms in Bioinformatics*, Dagstuhl, Germany: Schloss Dagstuhl – Leibniz-Zentrum für Informatik, 2024, 17:1–17:25.

[btaf104-B20] Levenshtein V. Binary codes capable of correcting deletions, insertions, and reversals. Soviet Physics-Doklady 1966;10:707–10.

[btaf104-B21] Li H. New strategies to improve minimap2 alignment accuracy. Bioinformatics 2021;37:4572–4. 34623391 10.1093/bioinformatics/btab705 PMC8652018

[btaf104-B22] Li H , Feng X, Chu C. The design and construction of reference pangenome graphs with minigraph. Genome Biol 2020;21:265. 33066802 10.1186/s13059-020-02168-z PMC7568353

[btaf104-B23] Liao W-W , Asri M, Ebler J et al A draft human pangenome reference. Nature 2023;617:312–24. 37165242 10.1038/s41586-023-05896-x PMC10172123

[btaf104-B24] Logsdon GA , Rozanski AN, Ryabov F et al The variation and evolution of complete human centromeres. Nature 2024;629:136–45. 38570684 10.1038/s41586-024-07278-3 PMC11062924

[btaf104-B25] Louza F , Gog S, Telles P. Inducing enhanced suffix arrays for string collections. Theor Comput Sci 2017;678:22–39.

[btaf104-B26] Lukjancenko O , Wassenaar T, Ussery D. Comparison of 61 sequenced *Escherichia coli* genomes. Microb Ecol 2010;60:708–20. 20623278 10.1007/s00248-010-9717-3 PMC2974192

[btaf104-B27] Marçais G , Delcher AL, Phillippy AM et al MUMmer4: a fast and versatile genome alignment system. PLoS Comput Biol 2018;14:e1005944. 29373581 10.1371/journal.pcbi.1005944 PMC5802927

[btaf104-B28] Nakamura T , Yamada KD, Tomii K et al Parallelization of MAFFT for large-scale multiple sequence alignments. Bioinformatics 2018;34:2490–2. 29506019 10.1093/bioinformatics/bty121 PMC6041967

[btaf104-B29] O'Donnell S , Fischer G. MUM&Co: accurate detection of all SV types through whole-genome alignment. Bioinformatics 2020;36:3242–3. 32096823 10.1093/bioinformatics/btaa115

[btaf104-B30] Olbrich J , Ohlebusch E, Büchler T. Generic non-recursive suffix array construction. ACM Trans Algorithms 2024;20:1–42.

[btaf104-B31] Olbrich J , Büchler T, Olebusch E. Generating multiple alignments of genomes of the same species. In: *Proceedings of the 18th International Joint Conference on Biomedical Engineering Systems and Technologies, Porto, Portugal*, Setúbal, Portugal: SciTePress, Vol. 1, 2025, 459–68.

[btaf104-B32] Porubsky D , Ebert P, Audano PA et al; Human Genome Structural Variation Consortium. Fully phased human genome assembly without parental data using single-cell strand sequencing and long reads. Nat Biotechnol 2021;39:302–8. 33288906 10.1038/s41587-020-0719-5 PMC7954704

[btaf104-B33] Puglisi S , Smyth W, Turpin A. A taxonomy of suffix array construction algorithms. ACM Comput Surv 2007;39:4.

[btaf104-B34] Tang F , Chao J, Wei Y et al HAlign 3: fast multiple alignment of ultra-large numbers of similar DNA/RNA sequences. Mol Biol Evol 2022;39:msac166. 35915051 10.1093/molbev/msac166 PMC9372455

[btaf104-B35] Tettelin H , Masignani V, Cieslewicz MJ et al Genome analysis of multiple pathogenic isolates of streptococcus agalactiae: implications for the microbial pan-genome. Proc Natl Acad Sci USA 2005;102:13950–5. 16172379 10.1073/pnas.0506758102 PMC1216834

[btaf104-B36] Treangen TJ , Ondov BD, Koren S et al The harvest suite for rapid core-genome alignment and visualization of thousands of intraspecific microbial genomes. Genome Biol 2014;15:524. 25410596 10.1186/s13059-014-0524-x PMC4262987

[btaf104-B37] Zhang P , Liu H, Wei Y et al FMAlign2: a novel fast multiple nucleotide sequence alignment method for ultralong datasets. Bioinformatics 2024;40:btae014. 38200554 10.1093/bioinformatics/btae014 PMC10809904

