# Supplementary material for: Generating multiple alignments on a pangenomic scale
Source: Bioinformatics. 2025 Mar 17;41(3):btaf104. doi: 10.1093/bioinformatics/btaf104 (PMC11928754; doi:10.1093/bioinformatics/btaf104)
Supplement: btaf104_Supplementary_Data [file btaf104_supplementary_data.pdf]

Supplementary material

Enumeration of lcp-intervals

Kasai et al. [2001] presented a linear time algorithm to simulate the bottom-up traversal of a suffix tree with a suffix array and its LCP-array (which, given the suffix array, can be constructed in linear time). The following algorithm is a slight modification of their algorithm TraverseWithArray, cf. [Abouelhoda et al., 2004]. It computes all lcp-intervals of the LCP-array with the help of a stack. The elements on the stack are lcp-intervals represented by tuples $\langle lcp, lb, rb\rangle$, where lcp is the lcp-value of the interval, lb is its left boundary, and rb is its right boundary. In Algorithm 1, push (pushes an element onto the stack) and pop (pops an element from the stack and returns that element) are the usual stack operations, while top provides a pointer to the topmost element of the stack. Furthermore, $\perp$ stands for an undefined value. We assume that array indexing starts at 1 and that $\mathsf{LCP}[1] = -1 = \mathsf{LCP}[n+1]$.

Algorithm 1 Given the LCP-array of a string of length n, this algorithm enumerates all lcp-intervals.

```
function ENUMERATE(LCP)
    push(⟨0, 1, ⊥⟩)
    for k ← 2 to n + 1 do
        lb ← k − 1
        while LCP[k] < top().lcp do
            top().rb ← k − 1
            interval ← pop()
            report(interval)
            lb ← interval.lb
        end while
        if LCP[k] > top().lcp then
            push(⟨LCP[k], lb, ⊥⟩)
        end if
    end for
end function
```

Proof of Lemma 2

Lemma 2 There is a one-to-one correspondence between the set of all `multiMUMs` and the set of all lcp-intervals ℓ-$[lb..rb]$ in the enhanced GSA of $S^1, \ldots, S^m$ satisfying

(1) $rb - lb + 1 = m$.

(2) $\mathsf{DA}[i] \neq \mathsf{DA}[j]$ for all pairs (i, j) with $lb \leq i < j \leq rb$.

(3) $\mathsf{BWT}[i] \neq \mathsf{BWT}[j]$ for at least one pair (i, j) with $lb \leq i < j \leq rb$.

Proof Let ℓ-$[lb..rb]$ be an lcp-interval satisfying the three conditions. By conditions (1) and (2), we have $\{\mathsf{DA}[k] \mid 1 \leq k \leq m\} = \{1, \ldots, m\}$. That is, the m suffixes in $[lb..rb]$ belong to m different strings. Let $i_1, \ldots, i_m$ be the permutation of the indices $lb, lb+1, \ldots, rb$ so that $\mathsf{DA}[i_k] = k$ for $1 \leq k \leq m$. Define $p_k = \mathsf{SA}[i_k]$ for $1 \leq k \leq m$. We claim that $(\ell, p_1, \ldots, p_m)$ is a `multiMUM`. By the definition of an lcp-interval, the length ℓ string $\omega = S^k[p_k..p_k + \ell - 1]$ is a common prefix of the suffixes in $[lb..rb]$. It follows that ω occurs exactly once in each of the strings $S^1, \ldots, S^m$ and that $(\ell, p_1, \ldots, p_m)$ is a multiple exact match. By condition (3), it is left-maximal. Since there is at least one index q in $[lb..rb]$ so that $\mathsf{LCP}[q] = \ell$ (definition of lcp-interval), it is also right-maximal. In summary, $(\ell, p_1, \ldots, p_m)$ is a `multiMUM`.

Conversely, let $(\ell, p_1, \ldots, p_m)$ be a `multiMUM`. That is, the string $\omega = S^k[p_k..p_k + \ell - 1]$ $(1 \leq k \leq m)$ occurs exactly once in each sequence $S^1, \ldots, S^m$. In combination with the right-maximality this implies that there is an lcp-interval ℓ-$[lb..rb]$ that contains exactly the suffixes $S^1_{p_1}, \ldots, S^m_{p_m}$. This lcp-interval satisfies conditions (1) and (2). It also satisfies condition (3) because $(\ell, p_1, \ldots, p_m)$ is left-maximal. □

An alternative method for computing multiMUMs

An alternative method for computing `multiMUMs` can be found in [Ohlebusch and Kurtz, 2008]. It works by (a) separately streaming each string S^j $(2 \leq j \leq m)$ against the suffix tree of S^1 and (b) combining the pairwise exact matches to multiple exact matches. In fact, the method described in [Ohlebusch and Kurtz, 2008] computes rare `multiMEMs`, but it yields `multiMUMs` if the rareness-thresholds are all set to 1.

Mumemto

Mumemto[1] is a program that also uses PFP to find `multiMUMs` (or `multiMEMs`), but it does not produce alignments [Shivakumar and Langmead, 2025]. Mumemto identifies `multiMUMs` on the base-level, while we do on the parse-level. It is thus not surprising that Mumemto is significantly slower and uses more memory than our program needs for finding the `multiMUMs`: For the 1000 Chromosome 19 haplotypes, Mumemto used 18 935s of CPU time and 39.9 GiB of RAM while we only used 3 512s of CPU time (59% of which is spent on parsing and a further 37% on computing the GSA of the parse) and 4.4 GiB of RAM.

Experiments with Mus musculus

We also tested PANAMA on 16 assemblies of Chromosome 1 (including the reference GRCm39 and assemblies of M. m. musculus, M. m. molossinus and M. m. castaneus).[2] The results can be seen in Table 6. It is noteworthy that the longest assembly in our dataset is 15% longer than the shortest one. Note that the running time is larger than one would expect considering Table 1. This seems to be due to one specific gap for which FMAlign2 runs unusually long.

Table 6. Evaluation of the generated alignments of 16 Chromosome 1 assemblies of Mus musculus. The rows have the same meaning as in Table 2. Note that all bases are included in the final alignment.

modulus	100	20
# anchors after 2c	247 812	883 991
# extensions at 2d	692 094	1 330 453
coverage in percent:		
after 2c (chaining)	11.57	36.63
after 2d (extension)	42.60	71.16
identity in percent:	66.69	66.67
running time (min)	85	81

[1] https://github.com/vikshiv/mumemto, last accessed 18.11.2024

[2] https://www.ncbi.nlm.nih.gov/datasets/genome/?taxon=10090&typical_only=true&assembly_level=3:3, last accessed: 18.11.2024

Experiments with simulated data

In order to test PANAMA on datasets with a known number of variations, we employed simuG[3] [Yue and Liti, 2019] to simulates the evolution of DNA sequences. The software-tool simuG simulates the full-spectrum of genomic variants: single nucleotide polymorphisms (SNPs), insertions/deletions (INDELS), copy number variants (CNV), inversions (INV), and translocations (TL). Three datasets were generated by simuG. Each dataset contains the sequence of chromosome 19 from the individual HG00096 as well as nine simulated offspring sequences. Each of the sequences has an approximate length of $55 \cdot 10^6$ bp. Each offspring was generated independently by adding random variations to HG00096. Except for the number of variations, simuG was executed with default parameters. In test set 1, the number of variations between an offspring and HG00096 is similar to real world data. However, we must multiply this number by 9 to get the total number of variations in the test set, because the offspring sequences were generated independently of each other. Consequently, the total number of variations in the test set is larger than in real human data. The simulated sequences in the test sets 2 and 3 contain twice and four times as many variants, respectively. Note that the generation of test set 3 already required over 13 hours. (When the number of variants was doubled once again, it was not possible to simulate a single sequence within 40 hours.) The results of our experiments with simulated data can be found in Table 7, which shows that PANAMA was able to efficiently generate a multiple alignment of good quality for the first test set. For the second test set, PANAMA also produces an alignment in short time, although less than 50% of the nucleotides were contained in blocks. When PANAMA was applied to the third test set, only 12% of the nucleotides were contained in blocks. This leaves huge gaps that need to be aligned externally with FMAlign2 and MAFFT. Although PANAMA is still able to generate an alignment, its time consumption increases substantially.

Table 7. Experiments with simulated data

The upper part of the table shows the number of variations in each of the nine offspring sequence in the test set. The middle part shows the percentage of nucleotides contained in blocks after Phase 2d, the number of 100% identity columns (i.e. columns without gaps in which all bases are identical) relative to the alignment length, and the sum of pairs score of the final alignment. The last row contains the running times of PANAMA.

test set	1	2	3
# SNPs	50,000	100,000	200,000
# INDELS	5,000	10,000	20,000
# CNV	5	10	20
# INV	5	10	20
# TL	5	10	20
nt in blocks (2d) in %	84.27	49.11	12.44
identities (final) in %	91.59	84.43	71.71
SP-value ($\times 10^6$)	3.14	6.17	12.6
time (min:sec)	1:39	3:26	33:19

[3] `https://github.com/yjx1217/simuG`, last accessed: 18.11.2024

References

M. Abouelhoda, S. Kurtz, and E. Ohlebusch. Replacing suffix trees with enhanced suffix arrays. *Journal of Discrete Algorithms*, 2(1):53–86, 2004.

T. Kasai et al. Linear-time longest-common-prefix computation in suffix arrays and its applications. In *Combinatorial Pattern Matching*, pages 181–192. Springer, 2001.

E. Ohlebusch and S. Kurtz. Space efficient computation of rare maximal exact matches between multiple sequences. *Journal of Computational Biology*, 15(4):357–377, 2008.

V. S. Shivakumar and B. Langmead. Mumemto: efficient maximal matching across pangenomes. *bioRxiv*, 2025.

J. Yue and G. Liti. simuG: a general-purpose genome simulator. *Bioinformatics*, 35:4442–4444, 2019.
